# Predicting Primary Refractory Diffuse Large B-Cell Lymphoma: A Comparison of Machine Learning Models with the IPI Score

**DOI:** 10.3390/jcm15176628

**Published:** 2026-08-27

**Authors:** Cosmin-Daniel Minciuna, Dorina Minciuna, Ingrid-Andrada Vasilache, Lucian Miron

**Affiliations:** 1 Grigore T. Popa University of Medicine and Pharmacy, 700115 Iasi, Romania; cosmin.minciuna@umfiasi.ro (C.-D.M.);; 2 Faculty of Medicine and Biological Sciences, ‘Ștefan cel Mare’ University, 720229 Suceava, Romania

**Keywords:** diffuse large B-cell lymphoma, primary refractory disease, machine learning, random forest, prediction model, calibration, decision curve analysis, SHAP, clinical risk score

## Abstract

**Background/Objectives**: Diffuse large B-cell lymphoma (DLBCL) remains clinically heterogeneous despite standard first-line immunochemotherapy. We evaluated whether machine learning (ML) models using routinely available diagnostic variables could predict primary refractory DLBCL. **Materials and Methods**: We performed a retrospective single-center study of 369 patients with DLBCL treated between 2015 and 2023. Primary refractory disease was defined as failure to achieve at least a partial response after first-line therapy. Logistic regression, random forest, gradient boosting, support vector machine with radial basis function kernel (SVM-RBF), and multilayer perceptron models were trained using baseline clinical and laboratory predictors. Performance was assessed using a stratified 80/20 holdout split, nested cross-validation, repeated random splits, and sensitivity analysis with complete hyperparameter retuning. **Results**: Primary refractory disease occurred in 111 patients (30.1%). In the holdout test set (*n* = 73), IPI achieved the highest discrimination (AUC 0.692; 95% CI 0.545–0.828). Among ML models, gradient boosting performed best (AUC 0.658; 95% CI 0.503–0.801), followed by SVM-RBF (AUC 0.619; 95% CI 0.471–0.760). DeLong comparisons showed no significant differences between ML models and IPI. In internal validation, gradient boosting showed the highest nested CV AUC (0.712 ± 0.049). Feature importance identified IPI as the dominant predictor. A weighted ML-derived clinical score identified a high-risk group with the highest refractory rate (52.9%), although the trend was not statistically significant (*p* = 0.063). **Conclusions**: ML models showed comparable but not superior performance to IPI for predicting primary refractory DLBCL. These findings support further external validation and development of multimodal clinically interpretable prediction frameworks for risk-adapted management.

## 1. Introduction

Diffuse large B-cell lymphoma (DLBCL) is consistently described as a frequent and aggressive malignancy and is the most common histologic subtype of non-Hodgkin lymphoma (NHL) [1,2,3]. DLBCL accounts for approximately 25% of adult NHL cases, and an estimated U.S. incidence of 6.9 new cases per 100,000 people per year has been reported [2], thus this data indicates substantial population burden and the potential impact of scalable clinical prediction tools.

First-line therapy for DLBCL commonly combines an anti-CD20 monoclonal antibody with anthracycline-based chemotherapy, most often delivered as R-CHOP (rituximab, cyclophosphamide, doxorubicin, vincristine, prednisone/prednisolone) [4]. With this approach, approximately 60–70% of patients can be cured, yet the remaining 30–40% experience relapse or refractoriness and frequently have poor outcomes, leaving an important unmet need for improved upfront risk stratification and treatment personalization [4,5].

Primary refractory disease represents a particularly high-risk form of treatment failure that is repeatedly highlighted as a significant challenge in DLBCL management due to poor prognosis. The overall fraction of patients experiencing relapse or refractory disease is commonly summarized in the 30–40% range, illustrating the magnitude of first-line treatment failure even in modern practice [6]. In addition, one study partitions this burden into 15% with primary refractory disease and 40% with relapse and refractory disease overall (11.8% relapse, 22.6% refractory after R-CHOP) [7], aligning with the broader relapse/refractory estimates and reinforcing the clinical relevance of predicting upfront failure rather than only relapse after response.

Because the definition of refractoriness determines both outcome ascertainment and model transportability, prediction modelling benefits from a clear operational endpoint. One clinically used definition identifies refractory patients as those with progressive or stable disease during first-line therapy, or failure to achieve at least a partial response after four or more cycles of immunochemotherapy [8,9,10]. Related early-failure endpoints such as POD24 incorporate primary refractory disease and relapse within two years, reflecting the persistent emphasis on early treatment failure as a marker of high-risk biology and adverse outcomes [11].

Risk stratification in DLBCL has traditionally relied on clinical scores such as the International Prognostic Index (IPI) because of their simplicity and convenience, and IPI-based tools remain the cornerstone of clinical assessment at diagnosis [12]. However, these indices make inadequate use of available clinical data, and multiple analyses have demonstrated that they may fail to consistently identify patients with poor outcomes, leaving substantial room for methodological improvement [13].

In the rituximab era, discriminative performance has been described as suboptimal, with reported c-indexes around 0.66 for IPI and 0.68 for NCCN-IPI in population-based cohorts, and the discriminatory power of IPI has been noted to diminish particularly in intermediate-risk groups after rituximab’s therapeutic impact [14,15]. A geriatric-adapted model incorporating functional and biochemical parameters has further demonstrated that IPI, R-IPI, and NCCN-IPI are outperformed in older patient cohorts [16]. More broadly, clinical outcome models based on pre-treatment features have been criticized for not identifying the molecular basis of clinical heterogeneity or specific therapeutic targets, consistent with DLBCL’s biological diversity and the clinical challenge of selecting personalized therapy strategies at diagnosis [17].

Machine learning (ML) has been proposed as a response to these limitations, given that ML methods can model complex, nonlinear interactions among variables without the distributional assumptions inherent to Cox proportional hazards regression and the log-rank test [14]. In DLBCL, this rationale is further strengthened by the availability of diverse data modalities not limited to clinical variables but also encompassing biological features, gene expression profiles, and imaging-based parameters including PET/CT radiomics that may refine estimates of relapse or refractory probability when available [18].

Accordingly, the aim of this study was to develop and internally validate supervised ML models for predicting primary refractory disease using routinely available baseline clinical variables, and to translate the most clinically relevant signals into a simplified risk score suitable for risk stratification and future prospective evaluation.

## 2. Materials and Methods

We conducted a retrospective, single-center study including DLBCL patients treated between 2015 and 2023. After deduplication and exclusion of records with ambiguous refractory-status coding, the final analytical cohort comprised 369 patients. The main outcome was primary refractory disease. Primary refractory DLBCL was defined as failure to achieve at least a partial response to first-line therapy, corresponding to stable disease or progressive disease during or by the end of first-line treatment.

We used baseline clinical and laboratory predictors routinely available at diagnosis, including age, sex, ECOG performance status, Ann Arbor stage, B symptoms, bulky disease, extranodal involvement, gastric involvement, histologic transformation, bone marrow infiltration, central nervous system involvement at diagnosis, IPI score, Ki-67 cutoff, LDH level, comorbidities, prior malignancy, viral hepatitis, and HIV status. CNS-IPI was excluded to avoid redundant prognostic information.

Ki-67 and LDH were available in the source database as predefined categories rather than continuous raw measurements. Ki-67 was coded as <75%, >75%, or unavailable, whereas LDH was categorized relative to the upper limit of normal (ULN) as normal, >ULN to <2 × ULN, 2 × ULN, 3 × ULN, or >3 × ULN. These categories reflected the predefined structure of the source database and were not derived or optimized according to refractory status. In the individual IPI-component framework, elevated LDH was defined as LDH above the ULN.

Three predictor frameworks were evaluated to assess the incremental predictive value of ML beyond IPI: (1) composite IPI plus non-IPI baseline predictors, (2) non-IPI predictors only, and (3) the five individual IPI components plus non-IPI predictors. Individual IPI components comprised age > 60 years, ECOG performance status ≥ 2, Ann Arbor stage III/IV, elevated LDH, and >1 extranodal site; the latter was reconstructed from the recorded anatomical extranodal sites.

Data were cleaned for duplicates and ambiguous refractory coding prior to modeling. For algorithms sensitive to predictor scaling (e.g., SVM and neural networks), predictors were standardized. We trained and compared five supervised learning approaches: logistic regression, random forest, gradient boosting machine, SVM with RBF kernel, and multilayer perceptron.

**Figure 1:**
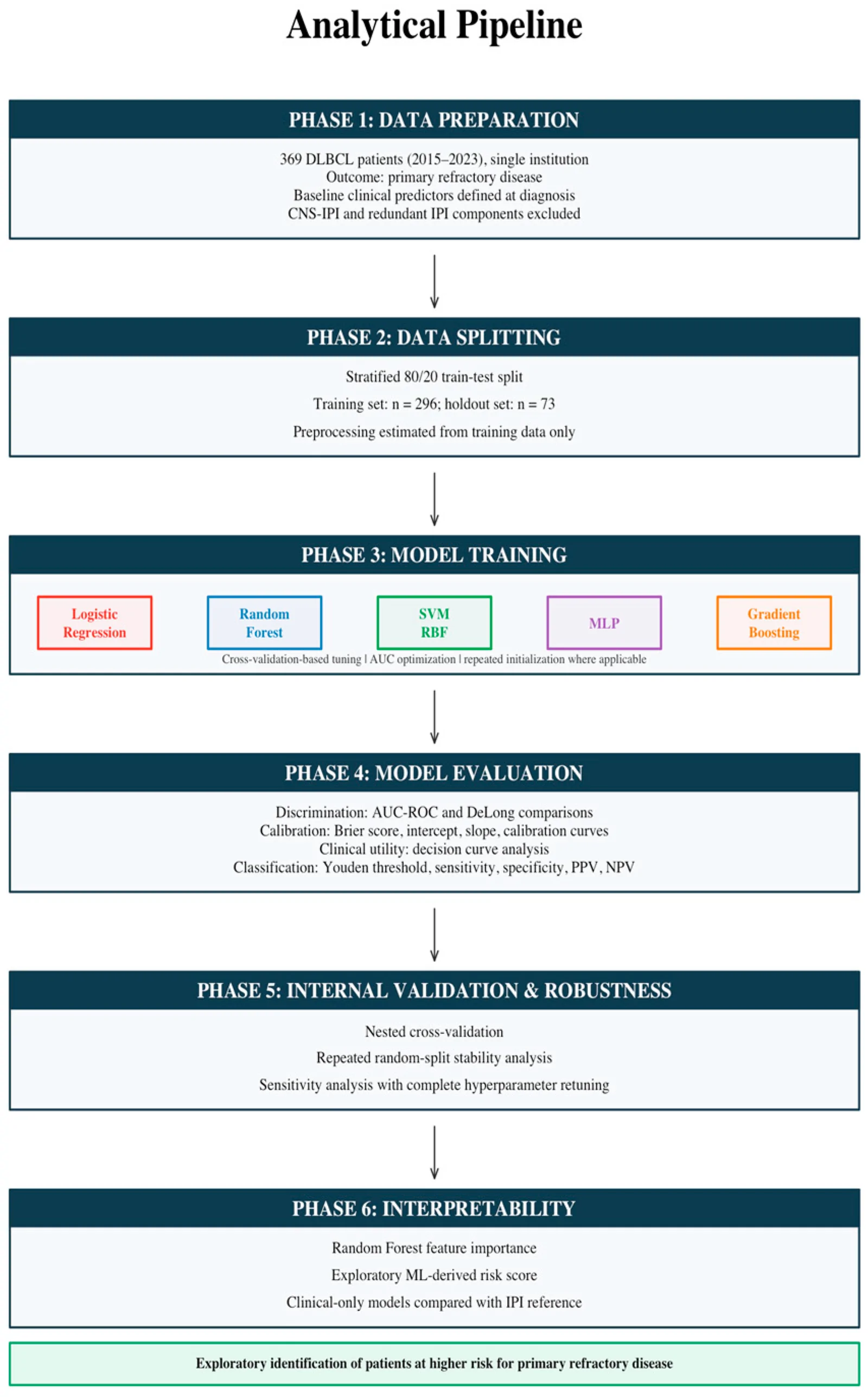
Analytical pipeline of model development and validation.

An analytical pipeline of model development and validation is presented in Figure 1. The complete patient-selection process, including exclusions related to unclassifiable first-line response status, ambiguous refractory-status coding, and duplicate removal, is presented in Supplementary Figure S1.

To limit overfitting, model tuning and performance estimation relied on resampling frameworks. We used a stratified 80/20 train–test split for primary holdout evaluation (Table 1). Internal validation additionally included nested cross-validation, repeated random-split stability analysis across 20 independent splits, and an internal sensitivity analysis using a separate stratified random split with complete hyperparameter retuning to assess robustness and expected generalization performance.

Discrimination was assessed using ROC analysis and AUC, consistent with guidance that prediction models are commonly evaluated by discrimination as part of overall predictive accuracy assessment. Pairwise comparisons between correlated ROC curves were performed using the DeLong test. The three IPI-based predictor frameworks were evaluated on the same holdout test set, with IPI alone serving as the reference.

Calibration was evaluated as the agreement between predicted probabilities and observed outcome frequencies across the spectrum of predicted risk. Calibration assessment included calibration curves, Brier score, calibration intercept, and calibration slope. Calibration intercept and slope were obtained using logistic recalibration models, with ideal values of 0 and 1, respectively.

In order to facilitate clinical interpretation at specific operating points, we summarized threshold-dependent classification metrics such as sensitivity and specificity, consistent with applied clinical ML reporting practices that present these metrics alongside discrimination and calibration summaries.

Clinical utility was assessed using decision curve analysis (DCA) which was interpreted via net benefit, plotted across threshold probabilities, with comparisons to default “treat all” and “treat none” strategies. Net benefit was conceptualized using the standard expression:

NB = (TP/n) − (FP/n) × (pt/(1 − pt)).

Feature importance was evaluated using complementary approaches including permutation importance, random forest Gini importance, and leave-one-out AUC reduction analysis. These methods were used to identify the most influential predictors contributing to refractory disease prediction.

We constructed an exploratory ML-derived clinical risk score using the most influential predictors identified from the random forest model. Predictor weights were proportional to normalized feature importance values and rescaled to generate an interpretable point-based score. Patients were subsequently stratified into low-, intermediate-, and high-risk groups according to score tertiles. All statistical analyses and machine-learning procedures were performed using R version 4.2.2 (R Foundation for Statistical Computing, Vienna, Austria). The R packages used were pROC version 1.18.0 for ROC/AUC estimation, confidence intervals, and DeLong testing; randomForest version 4.7-1.1 for random forest modeling; e1071 version 1.7-14 for SVM-RBF modeling; gbm version 2.1.9 for gradient boosting; nnet version 7.3-18 for multilayer perceptron modeling; rpart version 4.1-19 for tree-based utilities; and gridExtra version 2.3 together with base R graphics functions for figure generation.

## 3. Results

### 3.1. Patient Characteristics

A total of 369 patients with DLBCL were included in the analysis. Primary refractory disease was observed in 111/369 patients (30.1%), while 258/369 (69.9%) were classified as non-refractory. Baseline characteristics stratified by refractory status are summarized in Table 2.

In univariate analyses, patients with primary refractory disease had significantly higher IPI scores (*p* < 0.001), CNS-IPI scores (*p* < 0.001), and LDH levels (*p* < 0.001), as well as a higher proportion of male sex (57.7% vs. 43.0%, *p* = 0.012), worse ECOG performance status (*p* = 0.010), and more frequent bulky disease (46.8% vs. 31.0%, *p* = 0.004).

No statistically significant differences were observed for age at diagnosis (*p* = 0.134), Ki-67 (*p* = 0.547), Ann Arbor stage IV (*p* = 0.626), B symptoms (*p* = 0.169), extranodal involvement (*p* = 0.727), gastric involvement (*p* = 0.174), bone marrow infiltration (*p* = 0.825), or comorbidity burden (*p* = 0.526). The exact predictor specification, including source variables, coding, missingness, retained categories, preprocessing, and inclusion across the three predictor frameworks, is provided in Supplementary Table S1.

First-line treatment patterns differed significantly between non-refractory and primary refractory patients (Table 3). R-CHOP was the predominant regimen overall (270/369, 73.2%) but was less frequent in primary refractory than non-refractory patients (63.1% vs. 77.5%). The overall distribution of exact first-line regimens differed significantly by refractory status (*p* = 0.002).

This heterogeneity persisted after clinical aggregation of regimens (*p* < 0.001). A standard R-CHOP was used in 77.9% of non-refractory versus 63.1% of primary refractory patients, while non-rituximab-containing regimens were more frequent in the primary refractory group (9.9% vs. 0.4%).

Fewer than six cycles were administered to 52.3% of primary refractory versus 7.8% of non-refractory patients, while eight cycles were completed by 26.1% versus 68.6%, respectively (*p* < 0.001). Planned first-line therapy was completed in 33.3% of primary refractory compared with 96.1% of non-refractory patients (*p* < 0.001).

Radiotherapy exposure (10.8% vs. 8.1%, *p* = 0.429) and CNS relapse prophylaxis (8.1% vs. 8.5%, *p* = 0.894) did not differ significantly between primary refractory and non-refractory patients. Among the 13 patients with histologically transformed DLBCL, R-CHOP was the most frequent regimen (61.5%), followed by R-ADOx and GemOx (15.4% each) and R-CHEOP (7.7%). Regimen distribution did not differ significantly by refractory status in this subgroup (*p* = 0.307).

### 3.2. Model Discrimination on the Holdout Test Set

Model performance was evaluated on the stratified holdout test set comprising 73 patients (22 events and 51 non-events) (Table 4). The International Prognostic Index (IPI) demonstrated the highest discriminative ability (AUC 0.692, 95% CI 0.545–0.828), exceeding the performance of all evaluated machine learning models.

**Figure 2:**
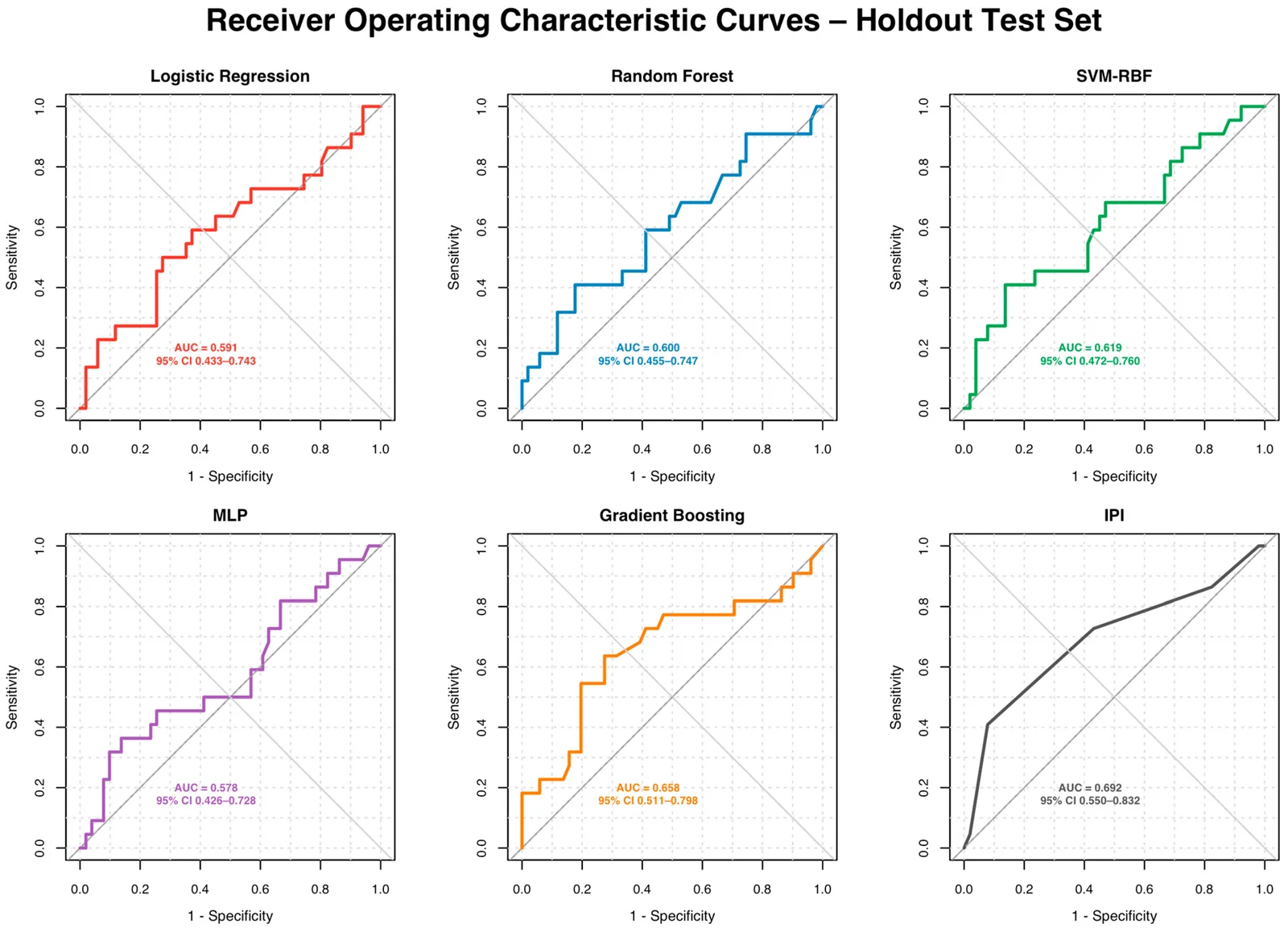
Receiver operating characteristic (ROC) curves for all models in the test set.

Among the ML approaches, Gradient Boosting achieved the best discrimination (AUC 0.658, 95% CI 0.503–0.801), followed by SVM-RBF (AUC 0.619, 95% CI 0.471–0.760), Random Forest (AUC 0.600, 95% CI 0.444–0.736), Logistic Regression (AUC 0.591, 95% CI 0.438–0.739), and MLP (AUC 0.578, 95% CI 0.425–0.723). A graphical representation of the ROC curves is shown in Figure 2.

Classification performance varied according to the selected decision threshold. Gradient Boosting achieved the highest sensitivity (63.6%), negative predictive value (82.2%), and F1 score (0.560), reflecting the most favorable balance between case detection and precision among the evaluated machine learning models (Table 3).

On the other hand, the IPI provided the highest specificity (92.2%), positive predictive value (69.2%), and overall accuracy (76.7%), albeit with lower sensitivity (40.9%). SVM-RBF also favored specificity (86.3%) over sensitivity (40.9%), whereas Random Forest, Logistic Regression, and MLP demonstrated intermediate performance across the evaluated classification metrics.

Calibration was generally acceptable across models. Gradient Boosting demonstrated calibration closest to the ideal, with a calibration intercept of 0.20 and a calibration slope of 1.00, while the IPI also showed good calibration (intercept 0.27, slope 1.19).

**Figure 3:**
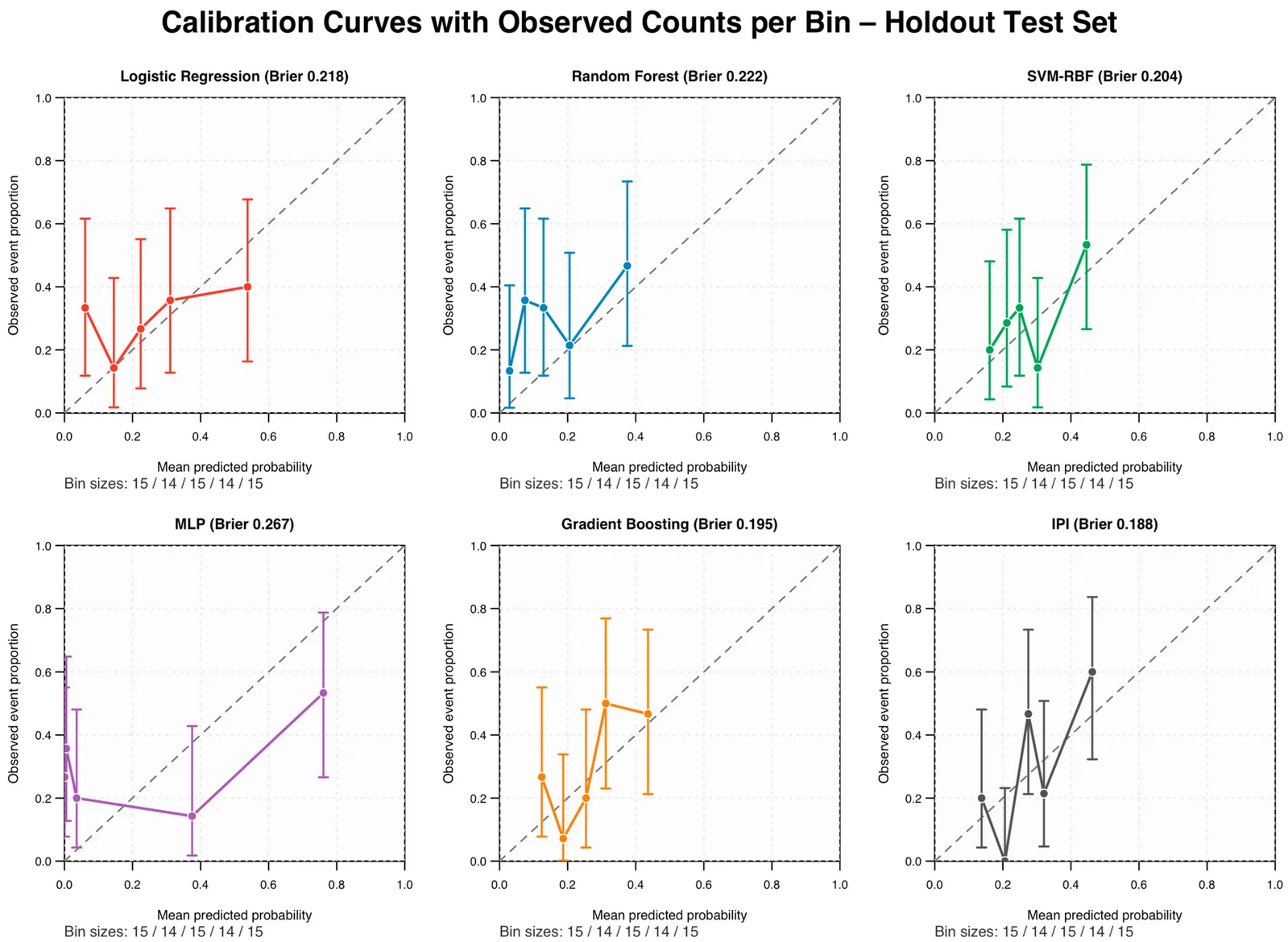
Calibration curves for all models in the test set.

In contrast, Logistic Regression, Random Forest, and MLP exhibited calibration slopes substantially below 1, indicating poorer agreement between predicted and observed risks. The IPI achieved the lowest Brier score (0.188), indicating the best overall probabilistic accuracy, whereas Brier scores for the machine learning models ranged from 0.195 to 0.267 (Table 4 and Figure 3).

Pairwise DeLong analyses demonstrated that none of the evaluated machine learning models differed significantly from the IPI with respect to discriminative performance (Table 5). The IPI achieved the highest AUC (0.692), whereas the machine learning models achieved AUCs ranging from 0.578 to 0.658.

The smallest difference relative to the IPI was observed for Gradient Boosting (ΔAUC = −0.034; Z = −0.686; *p* = 0.492), while MLP showed the largest reduction in discrimination compared with the IPI (ΔAUC = −0.114; Z = −1.608; *p* = 0.108). Logistic Regression also demonstrated lower discrimination than the IPI (ΔAUC = −0.101; Z = −1.679; *p* = 0.093), representing the comparison closest to statistical significance. None of these comparisons reached statistical significance.

Inter-model comparisons likewise revealed no statistically significant differences in discrimination. The largest numerical contrast was observed between Logistic Regression and Gradient Boosting (ΔAUC = −0.067; Z = −1.929; *p* = 0.054), favoring Gradient Boosting, although the difference did not reach statistical significance.

Logistic Regression and MLP exhibited nearly identical discriminative performance (ΔAUC = +0.012; *p* = 0.850), while Random Forest and SVM-RBF also showed comparable discrimination (ΔAUC = −0.019; *p* = 0.660). Similarly, Gradient Boosting and MLP demonstrated no significant difference in discriminative ability (ΔAUC = +0.080; *p* = 0.232).

### 3.3. Comparative Predictive Performance 

The comparison across predictor frameworks showed that none of the evaluated ML models improved discrimination over IPI alone (AUC 0.692, 95% CI 0.553–0.830) (Table 6). Models incorporating composite IPI achieved AUCs of 0.578–0.658, with Gradient Boosting performing best (AUC 0.658; ΔAUC = −0.034; *p* = 0.492). Exclusion of IPI markedly reduced discrimination (AUC 0.425–0.476), with all models performing significantly worse than IPI (all *p* ≤ 0.048). Models using individual IPI components showed intermediate performance (AUC 0.510–0.626), but none significantly outperformed IPI. Thus, the ML approaches provided no incremental discriminative value beyond IPI in the holdout cohort.

### 3.4. Classification Performance at the Youden Threshold

Classification metrics were evaluated at model-specific operating points derived from the Youden index (Table 7). At the selected classification thresholds, Gradient Boosting demonstrated the highest sensitivity (63.6%), negative predictive value (82.2%), and F1 score (0.560), indicating the most favorable balance between case detection and precision among the evaluated machine learning models.

On the other hand, the IPI achieved the highest specificity (92.2%), positive predictive value (69.2%), and overall accuracy (76.7%), reflecting superior performance in correctly identifying patients without the outcome while maintaining the best overall classification accuracy.

Among the remaining machine learning models, SVM-RBF and MLP both favored specificity (86.3%) over sensitivity (40.9% and 36.4%, respectively), whereas Random Forest showed slightly lower specificity (82.4%) with the same sensitivity as the IPI (40.9%). Logistic Regression provided an intermediate profile, achieving higher sensitivity (50.0%) at the expense of reduced specificity (72.5%).

### 3.5. Internal Validation of the Model

We performed three complementary internal validation procedures to evaluate model generalizability and robustness (Table 8). In nested cross-validation, Gradient Boosting achieved the highest mean discriminative performance (mean AUC 0.712 ± 0.049), followed by SVM-RBF (0.700 ± 0.066), Logistic Regression (0.692 ± 0.060), IPI (0.686 ± 0.075), MLP (0.683 ± 0.060), and Random Forest (0.680 ± 0.087). Random Forest exhibited the greatest variability across cross-validation folds (SD 0.087), whereas Gradient Boosting showed the lowest variability (SD 0.049), indicating the most consistent performance during nested cross-validation.

In the repeated split stability analysis, Gradient Boosting again achieved the highest mean AUC (0.685 ± 0.047), followed by the IPI (0.672 ± 0.053), SVM-RBF (0.666 ± 0.044), MLP (0.665 ± 0.050), Logistic Regression (0.663 ± 0.052), and Random Forest (0.651 ± 0.056). The relatively small standard deviations observed across all models indicate stable discriminative performance over repeated random train/test partitions.

In the sensitivity analysis with complete hyperparameter retuning, the IPI achieved the highest discrimination (AUC 0.717, 95% CI 0.591–0.830), followed closely by SVM-RBF (AUC 0.714, 95% CI 0.585–0.824), Gradient Boosting (AUC 0.709, 95% CI 0.577–0.835), Random Forest (AUC 0.702, 95% CI 0.564–0.824), MLP (AUC 0.689, 95% CI 0.545–0.821), and Logistic Regression (AUC 0.667, 95% CI 0.505–0.807).

### 3.6. Feature Importance of Random Forest 

**Figure 4:**
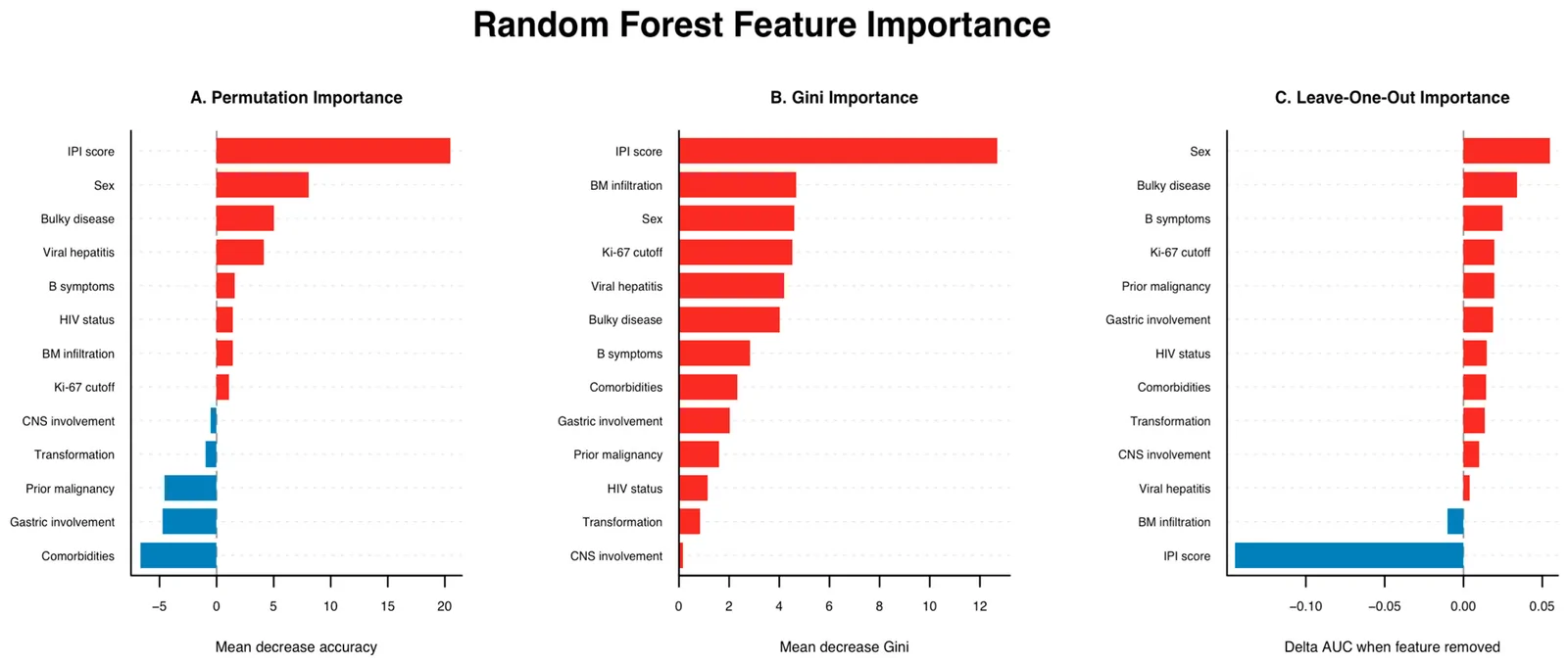
Feature importance.

Figure 4 presents complementary feature-importance analyses for the Random Forest model. Across the importance metrics, the composite IPI score emerged as the dominant predictor of primary refractory disease, confirming that established clinical risk burden accounted for the largest share of the model’s predictive signal. Additional contributors included sex, bulky disease, viral hepatitis, bone marrow infiltration, Ki-67 cutoff, and B symptoms, although their relative influence was substantially lower than that of IPI.

Permutation and Gini importance analyses both ranked IPI as the leading variable. Gini importance additionally highlighted bone marrow infiltration, sex, Ki-67 cutoff, viral hepatitis, and bulky disease as relevant secondary contributors. On the other hand, leave-one-out analysis showed a more heterogeneous pattern in which removal of some lower-ranked variables slightly improved AUC.

### 3.7. ML-Derived Clinical Risk Score

We developed an exploratory ML-derived clinical score using the most influential predictors identified by the Random Forest model (Table 9). In the holdout set, the weighted score stratified patients into low-, intermediate-, and high-risk groups, with refractory disease rates of 24.2%, 21.7%, and 52.9%, respectively. Although the high-risk group showed the highest event rate, the overall trend did not reach conventional statistical significance in the holdout cohort (Cochran-Armitage *p* = 0.063).

**Figure 5:**
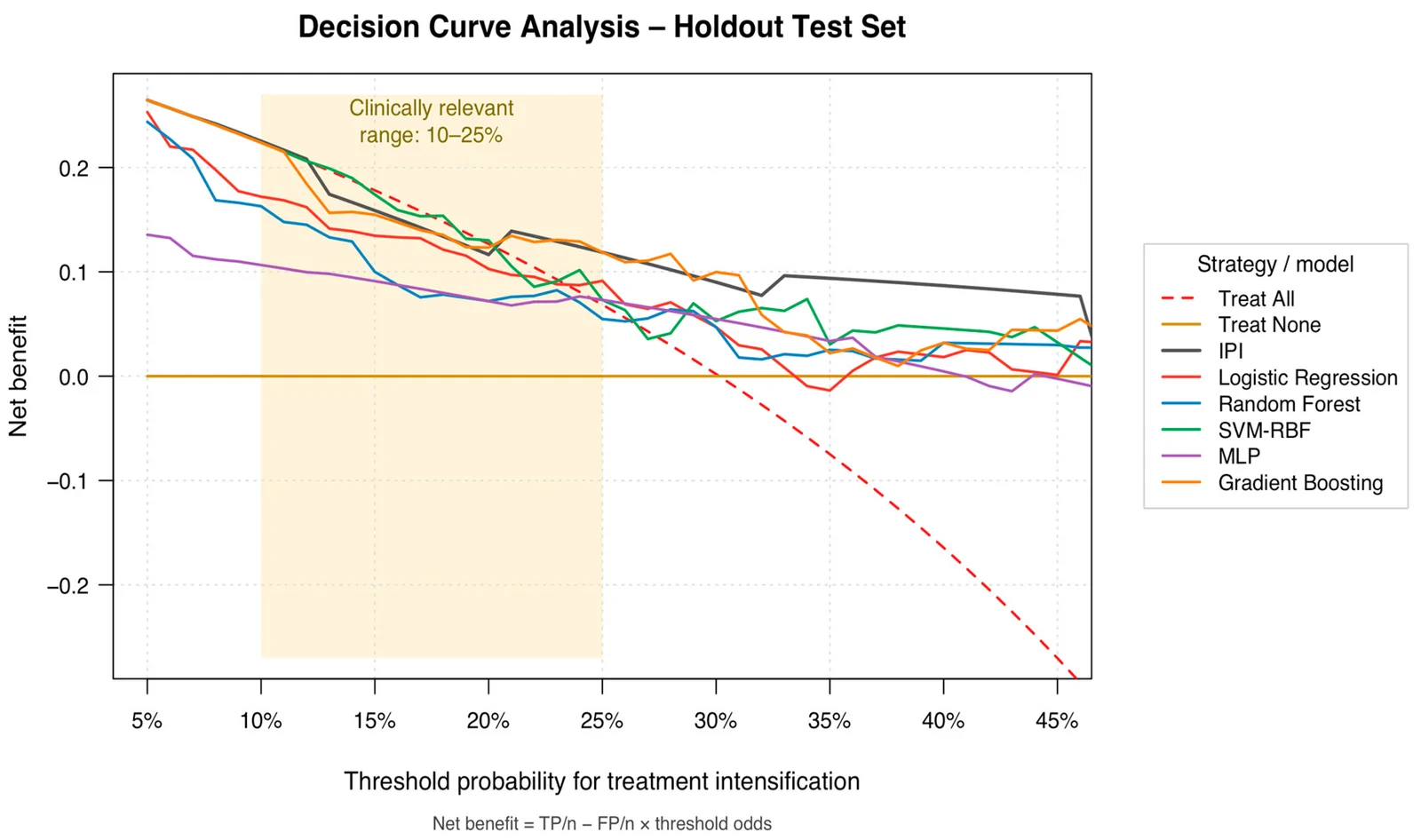
Decision curve analysis comparing clinical net benefit.

Decision curve analysis demonstrated that the evaluated models provided positive net clinical benefit across the clinically relevant threshold range of 10–25% (Figure 5). Within this interval, Gradient Boosting and SVM-RBF showed net benefit curves broadly comparable to the IPI reference model and to the treat-all strategy. However, the refitted holdout analysis did not support a consistent net-benefit advantage of all machine-learning models over IPI.

At lower thresholds around 10–15%, IPI, SVM-RBF, and Gradient Boosting showed the highest net benefit, while the treat-all strategy remained competitive because of the approximately 30% event prevalence in the holdout set. Around 20–25%, Gradient Boosting and IPI retained the most favorable net benefit among model-based approaches.

At higher threshold probabilities beyond approximately 30–35%, net benefit declined for all models and approached zero or negative values, indicating limited clinical gain for model-guided treatment intensification in high-threshold decision regions.

## 4. Discussion

Primary refractory DLBCL is clinically decisive because response to initial therapy is a dominant prognostic factor; primary refractory disease has been reported as strongly associated with inferior long-term outcomes, and achievement of durable response after initial treatment has been framed as central to lymphoma management. In this context, prediction models are developed to estimate an individual’s probability of an outcome occurring in the future to inform decision-making, including therapeutic planning and trial risk stratification. Consistent with these purposes, we developed and internally validated ML models to predict primary refractory disease using baseline clinical variables and translated these signals into a simplified risk score intended to support stratification rather than only reporting discrimination metrics.

The principal findings of this study were that: (i) IPI remained the strongest discriminator in the holdout test set; (ii) among ML models, Gradient Boosting and SVM-RBF showed the most favorable overall performance; (iii) model performance was moderate and broadly stable across internal validation procedures; and (iv) an exploratory ML-derived clinical score identified a high-risk subgroup, although the trend across risk strata did not reach statistical significance in the holdout cohort.

Compared with ML models, IPI remained the strongest discriminator in the holdout test set (AUC 0.692; 95% CI 0.545–0.828). Among ML approaches, Gradient Boosting achieved the highest holdout AUC (0.658; 95% CI 0.503–0.801), followed by SVM-RBF (AUC 0.619; 95% CI 0.471–0.760). DeLong analyses did not demonstrate statistically significant differences between any ML model and IPI. These findings suggest that routinely available clinical variables provide moderate predictive information, but ML models did not demonstrate robust incremental discrimination beyond established IPI-based risk assessment in this cohort.

Biccler et al. demonstrated, in a stacking approach applied to population-based Nordic registries encompassing over 5000 patients, that ML-based models achieved C-indexes of 0.756 (Denmark) and 0.744 (Sweden), substantially outperforming IPI (C-index Denmark, 0.662; Sweden, 0.661) and NCCN-IPI (Denmark, 0.681; Sweden, 0.681) [14]. Higher discriminative performance has been reported in studies incorporating high-dimensional inputs: random survival forest models integrating clinical and gene expression data from GEO-derived cohorts have achieved C-indexes of 0.832 in training and 0.758 in validation [19], while the GOYA trial ML analysis (*n* = 1166) reported test C-indexes up to 0.71 for RSF versus 0.65 for IPI, with albumin, LDH, SPD, and age as dominant features by SHAP analysis [20].

Santiago et al. developed a CT-based radiomics RF model using imaging features extracted from baseline lymph node volumes and reported AUC values of 0.83 and 0.79 for two independent readers, with sensitivity of 62% and specificity of 82% for distinguishing refractory from non-refractory patients [21]. These values were higher than the performance observed in our clinical-only models (best ML holdout AUC: 0.658 for Gradient Boosting), supporting the concept that incorporation of high-dimensional imaging features may improve predictive discrimination beyond routinely available clinical variables alone.

Internal validation demonstrated moderate but relatively stable discriminative performance across repeated analyses. Gradient Boosting achieved the highest nested cross-validation AUC (0.712 ± 0.049) and the highest repeated-split stability AUC (0.685 ± 0.047), whereas the sensitivity analysis showed comparable performance among IPI, SVM-RBF, Gradient Boosting, and Random Forest. These results indicate that model rankings varied across validation procedures and that no ML model showed consistent superiority over IPI.

Feature importance analyses consistently identified the composite IPI score as the dominant predictor across permutation, Gini, and leave-one-out analyses. Additional contributors included sex, bulky disease, viral hepatitis, bone marrow infiltration, Ki-67 cutoff, and B symptoms. This pattern suggests that the ML framework remained primarily anchored in established clinical risk captured by IPI, with only modest incremental contribution from individual baseline variables.

The exploratory ML-derived clinical score identified a high-risk subgroup with the highest observed refractory rate. In the holdout set, refractory disease occurred in 24.2% of low-risk, 21.7% of intermediate-risk, and 52.9% of high-risk patients. Although the high-risk group showed clear enrichment for primary refractory disease, the overall trend across risk categories did not reach conventional statistical significance (Cochran-Armitage *p* = 0.063). Therefore, this score should be considered exploratory and hypothesis-generating rather than ready for clinical implementation.

Decision curve analysis showed positive net benefit for several approaches across the clinically relevant threshold range of approximately 10–25%. Within this interval, Gradient Boosting and SVM-RBF showed net benefit curves broadly comparable to IPI and to the treat-all strategy. However, the holdout analysis did not support a consistent net-benefit advantage of ML models over IPI. At threshold probabilities above approximately 30–35%, net benefit declined for all models and approached zero or negative values, indicating limited clinical gain for model-guided treatment intensification in higher-threshold decision regions.

Werling et al., using registry data from 14,832 patients in the Danish DALY-CARE cohort, developed the MLAll model for prediction of treatment failure within 2 years of first-line therapy [22]. Compared with NCCN-IPI, MLAll improved recall from 0.32 to 0.39, precision from 0.56 to 0.60, and precision-recall AUC from 0.46 to 0.59, demonstrating that ML approaches can improve individualized risk stratification beyond traditional prognostic indices [22]. In contrast, in our smaller single-center cohort, ML models did not outperform IPI on holdout evaluation, underscoring the importance of sample size, endpoint definition, and external validation when applying ML to clinical-only baseline data.

Comparable observations were reported by Peng et al., who developed a molecularly integrated prognostic model (McPM) combining clinical and molecular variables using random survival forest and Bi-LSTM approaches [23]. Their model demonstrated superior ROC performance, C-index, and integrated Brier score compared with IPI for both overall and progression-free survival prediction [23]. They further derived a simplified clinically applicable score (sMcPM) incorporating extranodal involvement, LDH, MYC status, monocyte count, and platelet count. This parallels our approach of translating ML-derived feature importance into a simplified clinical score, supporting the concept that clinically interpretable reduced models may preserve substantial prognostic value while improving real-world applicability.

In the LymForest-25 study, Mosquera-Orgueira et al. validated a machine learning model integrating clinical, biochemical, and gene-expression variables, and demonstrated a substantially improved survival prediction compared with conventional cell of origin classification and molecular high-grade classifications [24]. Also, in the cited study, the integration of molecular signatures with IPI produced the best prognostic performance, reinforcing our observation that established clinical risk captured by IPI remains a dominant backbone even within more complex ML frameworks.

Similarly, Ma et al. developed an immune risk score derived from tumor microenvironment immune-cell composition using CIBERSORT and ESTIMATE algorithms in 956 DLBCL patients [25]. Their combined nomogram integrating immune signatures with clinical covariates achieved a C-statistic of 0.76, outperforming both IPI and R-IPI (C-statistics 0.69 and 0.69, respectively). These findings highlight the potential value of multimodal ML approaches, whereas our clinical-only models showed performance comparable, but not superior, to IPI.

Also, in a recent multicenter study including 418 DLBCL patients, Wang et al. combined interim PET-CT parameters with NCCN-IPI to construct the iPET-NCCN-IPI model [26]. The integrated model demonstrated superior prognostic performance compared with conventional approaches, further supporting the concept that multimodal models combining clinical, imaging, and biological information may outperform clinical-only prediction strategies such as ours.

The originality of this study resides from its evaluation of whether routinely available diagnosis-time clinical variables, modeled using ML, provide incremental predictive information beyond IPI for primary refractory DLBCL.

Several limitations should be acknowledged. First, this was a retrospective single-center study with a relatively modest cohort size, increasing susceptibility to sampling variability and limiting statistical power for formal intermodel comparisons.

Second, although multiple internal validation procedures were performed, external validation in independent multicenter cohorts remains necessary before clinical implementation.

Third, the predictor set was intentionally restricted to routinely available baseline clinical variables and did not include molecular profiling, radiomics, interim PET, or circulating tumor DNA data, all of which may substantially improve predictive accuracy in future multimodal approaches.

Fourth, biological and molecular predictors were not systematically recorded. This limitation could constitute and explanation for the modest performance of ML models in comparison with IPI score, as primary refractory DLBCL is strongly influenced by biological, molecular, metabolic, and host-related factors.

Fifth, although the cohort included 111 refractory events, the sample size remains modest for training and tuning multiple ML algorithms, comparing several predictor frameworks, and deriving an ML-based risk score. Moreover, the holdout test set included only 22 refractory events, limiting the precision of discrimination, calibration, and comparative performance estimates. Thus, the models and the simplified score should be considered exploratory and require external validation in larger independent cohorts.

Sixth, although R-CHOP was the predominant regimen and most patients received rituximab-containing therapy, first-line treatment was not completely homogeneous and differed according to refractory status, representing a potential source of confounding.

Finally, given its modest discrimination and the non-significant trend across risk groups in the holdout cohort, the ML-derived score should be regarded as hypothesis-generating and requires further refinement and external validation before its potential clinical utility can be established.

### Future Perspectives

Future work should prioritize external validation, recalibration in independent populations, and integration of biological and imaging biomarkers into multimodal prediction frameworks. Prospective evaluation of whether ML-informed risk stratification could change therapeutic decisions or improve patient outcomes will ultimately determine clinical utility.

From a data perspective, incorporating multimodal predictors is a rational next step. Radiomics-based ML has been described as feasible for identifying primary refractory DLBCL a priori and as providing relatively high prediction accuracy beyond standard qualitative imaging characteristics, supporting continued exploration of imaging augmentation. In addition, interim PET and circulating tumor DNA (ctDNA) strategies could be investigated in order to refine response assessment and salvage primary progressive cases.

## 5. Conclusions

Machine learning models using routinely available baseline clinical variables showed moderate discrimination for prediction of primary refractory DLBCL but did not outperform IPI in the holdout test set.

Among ML approaches, Gradient Boosting and SVM-RBF showed the most favorable overall performance, while IPI remained a strong benchmark across discrimination, calibration, and clinical utility analyses.

An exploratory ML-derived clinical score identified a high-risk subgroup, but risk stratification did not reach statistical significance in the holdout cohort.

These findings support the feasibility of internally validated ML-assisted risk modeling in DLBCL, while emphasizing the need for external validation and integration of multimodal biomarkers before clinical implementation.

## Figures and Tables

**Table 1:** Split summary.

Set	N	Events-Primary Refractory	Non-Events (Non-Refractory)	Event Rate
Full analysis cohort	369	111	258	0.3008130
Training set	296	89	207	0.3006757
Holdout test set	73	22	51	0.3013699

**Table 2:** Baseline Patient Characteristics by Primary Refractory Status.

Variable	Non-Refractory (*n* = 258)	Primary Refractory (*n* = 111)	*p* Value
Continuous variables, median [IQR]			
Age at diagnosis, years	63.0 [53.3–69.0]	65.0 [55.0–72.5]	0.134
IPI score	2.0 [2.0–3.0]	3.0 [3.0–4.0]	<0.001
CNS-IPI score	2.0 [2.0–3.0]	3.0 [2.0–4.0]	<0.001
Ki-67 category	2.0 [2.0–3.0]	2.0 [2.0–3.0]	0.547
LDH level category	1.0 [1.0–2.0]	2.0 [2.0–4.0]	<0.001
Male sex	111 (43.0%)	64 (57.7%)	0.012
ECOG performance status			0.010
0	30 (11.6%)	9 (8.1%)	
1	141 (54.7%)	44 (39.6%)	
2	57 (22.1%)	34 (30.6%)	
3–4	30 (11.6%)	24 (21.6%)	
Ann Arbor stage IV	153 (59.3%)	70 (63.1%)	0.626
B symptoms	145 (56.2%)	71 (64.0%)	0.169
Bulky disease	80 (31.0%)	52 (46.8%)	0.004
Extranodal involvement	157 (60.9%)	70 (63.1%)	0.727
Gastric involvement	48 (18.6%)	14 (12.6%)	0.174
Histologic transformation	9 (3.5%)	4 (3.6%)	1.000
Bone marrow infiltration	39 (15.1%)	14 (12.6%)	0.825
CNS involvement at diagnosis	2 (0.8%)	0 (0.0%)	1.000
Comorbidities present	190 (73.6%)	78 (70.3%)	0.526
Prior malignancy	15 (5.8%)	7 (6.3%)	0.815
Viral hepatitis (any)	52 (20.2%)	21 (18.9%)	0.887
HIV positive	3 (1.2%)	2 (1.8%)	0.639

Legend: CNS—central nervous system; CNS-IPI—Central Nervous System International Prognostic Index; ECOG—Eastern Cooperative Oncology Group; HIV—human immunodeficiency virus; IPI—International Prognostic Index; IQR—interquartile range; LDH—lactate dehydrogenase. Ki-67 categories: 1, <75%; 2, >75%; 3, unavailable. LDH categories: 1, normal; 2, >ULN to <2 × ULN; 3, 2 × ULN; 4, 3 × ULN; 5, >3 × ULN.

**Table 3:** First-line treatment characteristics according to primary refractory status.

Characteristic	Overall (N = 369)	Non-Refractory (*n* = 258)	Primary Refractory (*n* = 111)	*p* Value
A. Exact first-line systemic regimen				
R-CHOP	270 (73.2)	200 (77.5)	70 (63.1)	0.002
R-CNOP	22 (6.0)	13 (5.0)	9 (8.1)	
Mini R-CHOP	20 (5.4)	15 (5.8)	5 (4.5)	
R-CHEOP	19 (5.1)	12 (4.7)	7 (6.3)	
CEOP	10 (2.7)	1 (0.4)	9 (8.1)	
R-COP	5 (1.4)	4 (1.6)	1 (0.9)	
R-HyperCVAD	5 (1.4)	3 (1.2)	2 (1.8)	
(DA) R-EPOCH	3 (0.8)	2 (0.8)	1 (0.9)	
R-CEOP	3 (0.8)	1 (0.4)	2 (1.8)	
R-miniCEOP	3 (0.8)	2 (0.8)	1 (0.9)	
R-ADOx (Richter)	2 (0.5)	2 (0.8)	0 (0.0)	
GemOx (Richter)	3 (0.8)	2 (0.8)	1 (0.9)	
Leukeran/prednisone	1 (0.3)	0 (0.0)	1 (0.9)	
R-CEP	1 (0.3)	0 (0.0)	1 (0.9)	
NHL-BFM 95	2 (0.5)	0 (0.0)	1 (0.9)	
R-CHOP ± tafasitamab/lenalidomide (clinical trial)	1 (0.3)	1 (0.4)	0 (0.0)	
B. Treatment characteristics				
First-line regimen category				
Standard R-CHOP backbone	271 (73.4)	201 (77.9)	70 (63.1)	<0.001
Rituximab-containing CHOP-like, attenuated, or anthracycline-substitute regimen	73 (19.8)	47 (18.2)	26 (23.4)	
Other intensive/specialized rituximab-containing regimen	8 (2.2)	5 (1.9)	3 (2.7)	
Non-rituximab-containing regimen	12 (3.3)	1 (0.4)	11 (9.9)	
Transformation-directed regimen	4 (1.1)	3 (1.2)	1 (0.9)	
Documented rituximab exposure	354 (95.9)	255 (98.8)	99 (89.2)	<0.001
Number of first-line cycles administered				
<6 cycles	78 (21.1)	20 (7.8)	58 (52.3)	<0.001
6 cycles	85 (23.0)	61 (23.6)	24 (21.6)	
8 cycles	206 (55.9)	177 (68.6)	29 (26.1)	
Completion of planned first-line therapy	285 (77.2)	248 (96.1)	37 (33.3)	<0.001
Radiotherapy exposure	33 (8.9)	21 (8.1)	12 (10.8)	0.429
CNS relapse prophylaxis	31 (8.4)	22 (8.5)	9 (8.1)	0.894
C. First-line systemic treatment in histologically transformed DLBCL				
	*n* = 13	*n* = 9	*n* = 4	
R-CHOP	8 (61.5)	6 (66.7)	2 (50.0)	0.307
R-ADOx (Richter)	2 (15.4)	2 (22.2)	0 (0.0)	
GemOx (Richter)	2 (15.4)	1 (11.1)	1 (25.0)	
R-CHEOP	1 (7.7)	0 (0.0)	1 (25.0)	

**Table 4:** Model Discrimination: Holdout Test Set Performance (*n* = 73 patients).

Model	AUC (95% CI)	Brier Score	Calibration Intercept	Calibration Slope	Threshold	Sensitivity	Specificity	PPV	NPV	Accuracy	F1 Score
IPI	0.692 (0.545–0.828)	0.188	0.272	1.189	0.391	0.409	0.922	0.692	0.783	0.767	0.514
Logistic Regression	0.591 (0.438–0.739)	0.218	−0.552	0.238	0.285	0.500	0.725	0.440	0.771	0.658	0.468
Random Forest	0.600 (0.444–0.736)	0.222	−0.307	0.274	0.232	0.409	0.824	0.500	0.764	0.699	0.450
SVM-RBF	0.619 (0.471–0.760)	0.204	−0.068	0.783	0.340	0.409	0.863	0.563	0.772	0.726	0.474
Gradient Boosting	0.658 (0.503–0.801)	0.195	0.199	0.995	0.279	0.636	0.725	0.500	0.822	0.699	0.560
MLP	0.578 (0.425–0.723)	0.267	−0.658	0.057	0.628	0.364	0.863	0.533	0.759	0.712	0.432

Legend: AUC—area under the receiver operating characteristic curve; CI—confidence interval; IPI—International Prognostic Index; MLP—multilayer perceptron; SVM-RBF—support vector machine with radial basis function kernel.

**Table 5:** DeLong Test.

**A. Models vs. IPI**					
**Comparison**	**AUC Model 1**	**AUC Model 2**	**Difference**	**Z Statistic**	***p* Value**
Logistic Regression vs. IPI	0.591	0.692	−0.101	−1.679	0.093
Random Forest vs. IPI	0.600	0.692	−0.092	−1.292	0.196
SVM-RBF vs. IPI	0.619	0.692	−0.074	−1.104	0.270
Gradient Boosting vs. IPI	0.658	0.692	−0.034	−0.686	0.492
MLP vs. IPI	0.578	0.692	−0.114	−1.608	0.108
**B. Inter-Model Comparisons**					
Logistic Regression vs. Random Forest	0.591	0.600	−0.009	−0.187	0.852
Logistic Regression vs. SVM-RBF	0.591	0.619	−0.028	−0.887	0.375
Logistic Regression vs. Gradient Boosting	0.591	0.658	−0.067	−1.929	0.054
Logistic Regression vs. MLP	0.591	0.578	+0.012	0.189	0.850
Random Forest vs. SVM-RBF	0.600	0.619	−0.019	−0.439	0.660
Random Forest vs. Gradient Boosting	0.600	0.658	−0.058	−1.122	0.262
Random Forest vs. MLP	0.600	0.578	+0.021	0.404	0.686
SVM-RBF vs. Gradient Boosting	0.619	0.658	−0.040	−0.912	0.362
SVM-RBF vs. MLP	0.619	0.578	+0.040	0.614	0.540
Gradient Boosting vs. MLP	0.658	0.578	+0.080	1.195	0.232

Abbreviations: AUC—area under the receiver operating characteristic curve; IPI—International Prognostic Index; MLP—multilayer perceptron; SVM-RBF—support vector machine with radial basis function kernel. DeLong tests were used for pairwise comparisons of correlated ROC curves. Positive differences indicate superior discrimination of the first-listed model.

**Table 6:** Comparative Predictive Performance Across IPI-Based Predictor Frameworks.

Predictor Framework	Model	AUC (95% CI)	ΔAUC vs. IPI	DeLong Z	*p* Value
Reference	IPI alone	0.692 (0.553–0.830)	—	—	—
Composite IPI + non-IPI predictors	Logistic Regression	0.591 (0.434–0.749)	−0.101	−1.679	0.093
	Random Forest	0.600 (0.446–0.737)	−0.092	−1.292	0.196
	SVM-RBF	0.619 (0.467–0.759)	−0.074	−1.104	0.270
	Gradient Boosting	0.658 (0.500–0.802)	−0.034	−0.686	0.492
	MLP	0.578 (0.422–0.721)	−0.114	−1.608	0.108
Non-IPI predictors only	Logistic Regression	0.443 (0.290–0.603)	−0.249	−2.464	0.014
	Random Forest	0.425 (0.283–0.572)	−0.267	−2.551	0.011
	SVM-RBF	0.476 (0.329–0.622)	−0.216	−1.977	0.048
	Gradient Boosting	0.439 (0.282–0.604)	−0.253	−2.459	0.014
	MLP	0.431 (0.279–0.586)	−0.261	−2.601	0.009
Individual IPI components + non-IPI predictors	Logistic Regression	0.585 (0.428–0.740)	−0.107	−1.702	0.089
	Random Forest	0.544 (0.394–0.695)	−0.148	−2.142	0.032
	SVM-RBF	0.535 (0.372–0.692)	−0.157	−2.115	0.034
	Gradient Boosting	0.626 (0.475–0.771)	−0.066	−1.027	0.304
	MLP	0.510 (0.360–0.663)	−0.182	−2.560	0.010

Legend: AUC, area under the receiver operating characteristic curve; CI, confidence interval; IPI, International Prognostic Index; MLP, multilayer perceptron; SVM-RBF, support vector machine with radial basis function kernel.

**Table 7:** Classification Performance at Optimal Youden Threshold (Test Set).

Model	Threshold	Sensitivity	Specificity	PPV	NPV	Accuracy	F1 Score
Logistic Regression	0.285	0.500	0.725	0.440	0.771	0.658	0.468
Random Forest	0.232	0.409	0.824	0.500	0.764	0.699	0.450
SVM-RBF	0.340	0.409	0.863	0.562	0.772	0.726	0.474
MLP	0.628	0.364	0.863	0.533	0.759	0.712	0.432
Gradient Boosting	0.279	0.636	0.725	0.500	0.822	0.699	0.560
IPI	0.391	0.409	0.922	0.692	0.783	0.767	0.514

Abbreviations: MLP—multilayer perceptron; NPV—negative predictive value; PPV—positive predictive value; SVM-RBF—support vector machine with radial basis function kernel.

**Table 8:** Internal Validation.

Model	Nested CV (Mean AUC ± SD)	Repeated Split Stability (Mean AUC ± SD)	Sensitivity Analysis AUC (95% CI)
Logistic Regression	0.692 ± 0.060	0.663 ± 0.052	0.667 (0.505–0.807)
Random Forest	0.680 ± 0.087	0.651 ± 0.056	0.702 (0.564–0.824)
SVM-RBF	0.700 ± 0.066	0.666 ± 0.044	0.714 (0.585–0.824)
MLP	0.683 ± 0.060	0.665 ± 0.050	0.689 (0.545–0.821)
Gradient Boosting	0.712 ± 0.049	0.685 ± 0.047	0.709 (0.577–0.835)
IPI	0.686 ± 0.075	0.672 ± 0.053	0.717 (0.591–0.830)

Abbreviations: AUC—area under the receiver operating characteristic curve; CI—confidence interval; CV—cross-validation; IPI—International Prognostic Index; MLP—multilayer perceptron; SD—standard deviation; SVM-RBF—support vector machine with radial basis function kernel.

**Table 9:** Risk stratification.

Risk Group	*n*	Refractory (%)	95% CI
Low risk	33	8 (24.2%)	11.1–42.3%
Intermediate risk	23	5 (21.7%)	7.5–43.7%
High risk	17	9 (52.9%)	27.8–77.0%
Test	Value	*p* value	
Chi-square	5.51	0.063	
Cochran–Armitage trend test	3.46	0.063	
AUC score:	0.619 (95% CI 0.448–0.776)		

Abbreviations: CI—confidence interval; AUC—area under the receiver operating characteristic curve.

## Data Availability

The datasets are available from the correspondent authors upon a reasonable request due to local policies.

## Supplementary Materials

The following supporting information can be downloaded at https://www.mdpi.com/article/10.3390/jcm15176628/s1, Figure S1—Patient flow; Supplementary Table S1. Predictor definitions, coding, missingness, and preprocessing across modeling frameworks.

## Abbreviations

The following abbreviations are used in this manuscript:

DLBCL	Diffuse large B-cell lymphoma
NHL	Non-Hodgkin lymphoma
R-CHOP	Rituximab, cyclophosphamide, doxorubicin, vincristine, prednisone/prednisolone
IPI	International Prognostic Index
NCCN-IPI	National Comprehensive Cancer Network International Prognostic Index
ML	Machine learning
PET/CT	Positron emission tomography/computed tomography
ECOG	Eastern Cooperative Oncology Group
CNS	Central nervous system
CNS-IPI	Central Nervous System International Prognostic Index
Ki-67	Ki-67 proliferation index
LDH	Lactate dehydrogenase
HIV	Human immunodeficiency virus
SVM	Support vector machine
SVM-RBF	Support vector machine with radial basis function kernel
RBF	Radial basis function
MLP	Multilayer perceptron
ROC	Receiver operating characteristic
AUC	Area under the receiver operating characteristic curve
CI	Confidence interval
IQR	Interquartile range
PPV	Positive predictive value
NPV	Negative predictive value
CV	Cross-validation
SD	Standard deviation
DCA	Decision curve analysis
NB	Net benefit
TP	True positive
FP	False positive
GEO	Gene Expression Omnibus
RF	Random forest
RSF	Random survival forest
CT	Computed tomography
McPM	Molecularly integrated prognostic model
sMcPM	Simplified molecularly integrated prognostic model
Bi-LSTM	Bidirectional Long Short-Term Memory
ESTIMATE	Estimation of STromal and Immune cells in MAlignant Tumor tissues using Expression data
