# Supplementary material for: Predicting Primary Refractory Diffuse Large B-Cell Lymphoma: A Comparison of Machine Learning Models with the IPI Score
Source: J Clin Med. 2026 Aug 27;15(17):6628. doi: 10.3390/jcm15176628 (PMC13566510; doi:10.3390/jcm15176628)

Patient Selection Flow

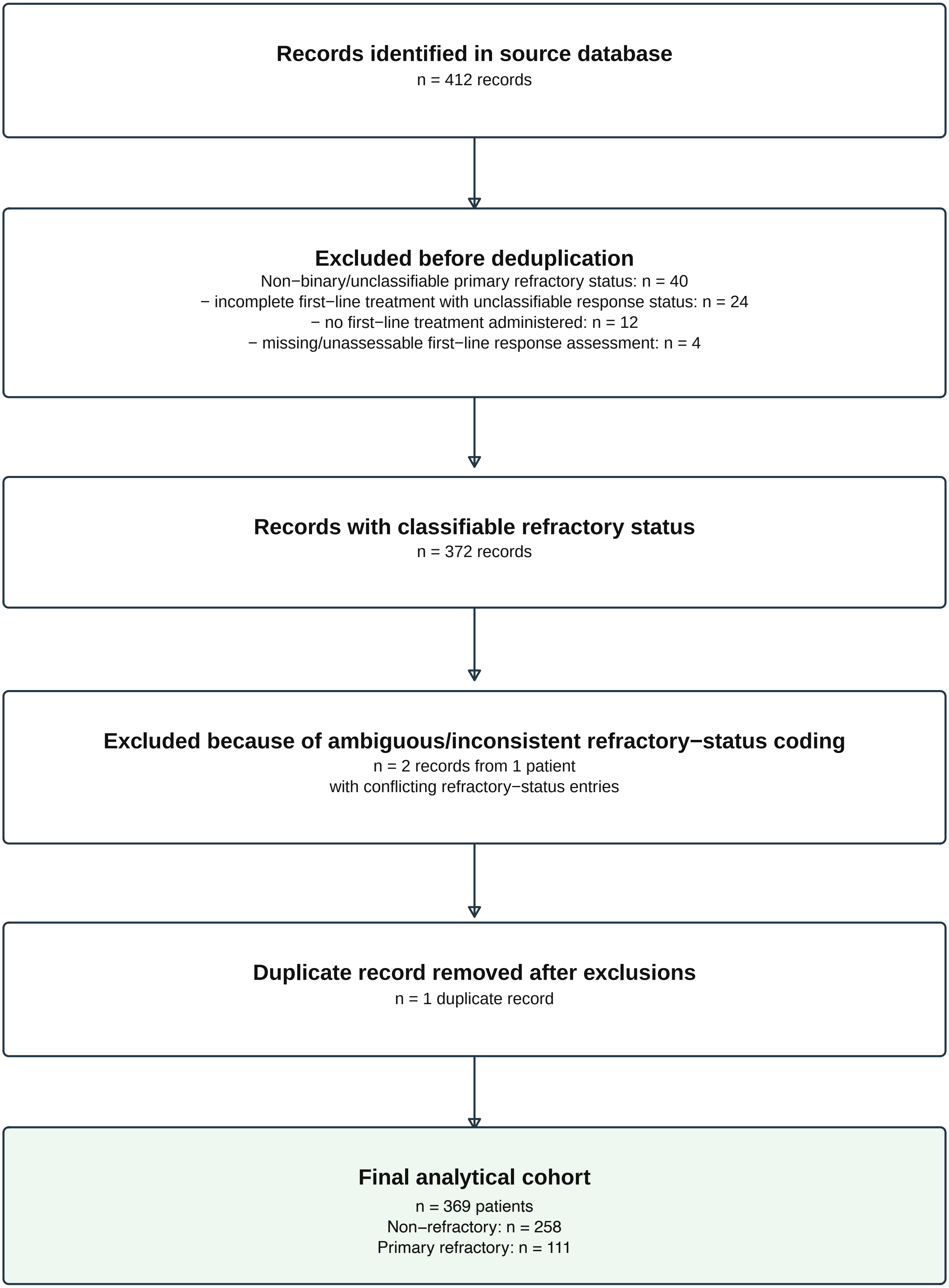

Supplement: Supplementary file 1 [file jcm-15-06628-s001.zip › Supp fig 1.pdf]
