# Supplementary material for: Predicting Primary Refractory Diffuse Large B-Cell Lymphoma: A Comparison of Machine Learning Models with the IPI Score
Source: J Clin Med. 2026 Aug 27;15(17):6628. doi: 10.3390/jcm15176628 (PMC13566510; doi:10.3390/jcm15176628)
Supplement: Supplementary file 1 [file jcm-15-06628-s001.zip › supplement limf.pdf]

Supplementary Table S1. Predictor definitions, coding, missingness, and preprocessing across modeling frameworks

Predictor	Source variable	Coding/categories used for modeling	Missing/unavailable, n (%)	Composite IPI + non-IPI	Non-IPI only	Individual IPI components + non-IPI	Preprocessing
Composite IPI score	IPI	0–5 ordinal score	0 (0.0%)	Yes	No	No	Numeric ordinal predictor; scaled for SVM-RBF/MLP
Sex	Sex	1 = male; 2 = female	0 (0.0%)	Yes	Yes	Yes	Categorical; factor/dummy encoding
B symptoms	Semne_B	1 = absent/A; 2 = present/B	0 (0.0%)	Yes	Yes	Yes	Categorical; factor/dummy encoding
Bulky disease	Bulky_disease	1 = no; 2 = yes	0 (0.0%)	Yes	Yes	Yes	Categorical; factor/dummy encoding
Gastric involvement	Gastric	1 = no; 2 = yes	0 (0.0%)	Yes	Yes	Yes	Categorical; factor/dummy encoding
Histologic transformation	Transformare	1 = no; 2 = yes	0 (0.0%)	Yes	Yes	Yes	Categorical; factor/dummy encoding
Bone marrow infiltration	Infiltrare_MO	1 = no; 2 = yes; 3 = not evaluated	224 (60.7%); code 3 retained	Yes	Yes	Yes	Categorical; code 3 retained as separate level
CNS involvement at diagnosis	Infiltrare_SNC_dg	1 = no; 2 = yes	0 (0.0%)	Yes	Yes	Yes	Categorical; factor/dummy encoding
Ki-67 cutoff	Ki67_cutoff	1 = <75%; 2 = >75%; 3 = no data	172 (46.6%); code 3 retained	Yes	Yes	Yes	Categorical; code 3 retained as separate level
Comorbidities	Comorbiditati	1 = no; 2 = yes	0 (0.0%)	Yes	Yes	Yes	Categorical;

							factor/dummy encoding
Prior malignancy	APP_neo	1 = no; 2 = yes	0 (0.0%)	Yes	Yes	Yes	Categorical; factor/dummy encoding
Viral hepatitis	Hepatita_virala	1 = no; 2 = HBV; 3 = HCV	0 (0.0%)	Yes	Yes	Yes	Categorical; factor/dummy encoding
HIV status	HIV	1 = no; 2 = yes	0 (0.0%)	Yes	Yes	Yes	Categorical; factor/dummy encoding
Age >60 years	Varsta_cuttof	Binary: 1 if >60 years, otherwise 0	0 (0.0%)	No	No	Yes	Scaled for SVM-RBF/MLP
ECOG ≥2	ECOG	Binary: 1 if ECOG ≥2, otherwise 0	0 (0.0%)	No	No	Yes	Scaled for SVM-RBF/MLP
Ann Arbor stage III/IV	Stadiu_dg	Binary: 1 if stage III/IV, otherwise 0	0 (0.0%)	No	No	Yes	Scaled for SVM-RBF/MLP
Elevated LDH	LDH	Binary: 1 if LDH category 2–5; 0 if category 1	0 (0.0%)	No	No	Yes	Scaled for SVM-RBF/MLP
>1 extranodal site	Tip_localizare_extra	Binary: 1 if yes.	0 (0.0%)	No	No	Yes	Scaled for SVM-RBF/MLP

Abbreviations: CNS, central nervous system; ECOG, Eastern Cooperative Oncology Group; HBV, hepatitis B virus; HCV, hepatitis C virus; HIV, human immunodeficiency virus; IPI, International Prognostic Index; LDH, lactate dehydrogenase; ML, machine learning; MLP, multilayer perceptron; SVM-RBF, support vector machine with radial basis function kernel.
